# Rheological, Chemical and Physical Characteristics of Golden Berry (*Physalis peruviana* L.) after Convective and Microwave Drying

**DOI:** 10.3390/foods6080060

**Published:** 2017-07-29

**Authors:** Agnieszka Nawirska-Olszańska, Bogdan Stępień, Anita Biesiada, Joanna Kolniak-Ostek, Maciej Oziembłowski

**Affiliations:** 1 Department of Fruit, Vegetable and Plant Nutraceutical Technology, Wroclaw University of Environmental and Life Sciences, str. Chełmońskiego 37, 51-630 Wroclaw, Poland; joanna.kolniak-ostek@upwr.edu.pl; 2 Institute of Agricultural Engineering, Wroclaw University of Environmental and Life Sciences, str. Chełmońskiego 41, 51-630 Wroclaw, Poland; bogdan.stepien@upwr.edu.pl; 3 Department of Horticulture, Wroclaw University of Environmental and Life Sciences, Plac Grunwaldzki 24a, 50-363 Wroclaw, Poland; anita.biesiada@upwr.edu.pl; 4 Department of Animal Products Technology and Quality Management, Wroclaw University of Environmental and Life Sciences, str. Chełmońskiego 37, 51-630 Wroclaw, Poland; maciej.oziemblowski@upwr.edu.pl

**Keywords:** microwave treatment under reduced pressure, bioactive compounds, colour, antioxidant activity

## Abstract

Studies on methods for fixing foods (with a slight loss of bioactive compounds) and obtaining attractive products are important with respect to current technology. The drying process allows for a product with highly bioactive properties. Drying of *Physalis* fruit was carried out in a conventional manner, and in a microwave under reduced pressure at 120 W and 480 W. After drying, the fruits were subjected to strength and rheological tests. Water activity, content of carotenoids and polyphenols and antioxidant activity as well as colour were also examined. The study showed that *Physalis* is a difficult material for drying. The best results were obtained using microwave drying at a power of 480 W. *Physalis* fruit microwave-dried by this method is characterized by higher resistance to compression than the fruit dried by convection. Dried fruit obtained in this way was characterized by higher contents of bioactive compounds, better antioxidant properties, and at the same time the lowest water activity.

## 1. Introduction

*Physalis peruviana* L. of the *Solanaceae* family, called Peruvian groundcherry or Brazilian raisin, is a cultivated and medicinal plant virtually unknown in Poland, procured fresh in small quantities and used for garnishing ice-creams and other confectionary products. Its common English names are Cape gooseberry (South Africa), Inca berry, Aztec berry, golden berry, giant ground cherry, Peruvian groundcherry, and sometimes, simply *Physalis* (UK). It originates from South America, and currently it is cultivated in California, Australia, New Zealand and Egypt, but also in Europe [1].

The groundcherry is a perennial, herbaceous plant growing up to 0.5–2.0 m high. Its edible parts are small (1–2 cm in diameter with mass of 4–10 g) bright orange fruits enveloped in a dry parchment husk of bright beige colour and lantern shape. The fruits are characterized by a very pleasant, refreshing, sour-sweet taste and pleasant aroma. A single fruit contains approximately 100–200 seeds. The berries are very durable and can be stored for a very long time: for over 3 months at room temperature, and as reported by Puente [2] at 2 °C for 5–6 months without signs of spoilage or wilting [3].

Although at the moment there are few publications in the world literature on the chemical composition of this plant, on the basis of available sources it can be concluded that the fruit of Brazilian raisin has a rich chemical composition and high nutritional value, and can also be used for the production of functional foods [4]. The dry matter content reaches 10.4–17.24%, 15% of which is composed of soluble substances. According to Rodrigues et al. [5], the content of total carbohydrates is 13.22%, total fats 3.16% and protein 1.85%. Its energy value is relatively high at 88.72 kcal. The content of total sugars is up to 8.9%, including 6.4% reducing sugars. Brazilian raisin contains much fructose, and may therefore be recommended for diabetics. Its vitamin C content varies from 38 to 43 mg% and in carotenes 1.6 mg%, containing 70% *trans* β-carotene [4]. It also has in its composition a vitamin B complex (thiamine, niacin and riboflavin), tocopherols (mainly γ- and α-), vitamin K, fibre and a very rich composition of macro- and micronutrients (with iron, zinc and copper) [5]. The fruit contains fatty acids, including a high proportion of polyunsaturated fatty acids (gamma linolenic acid constitutes 18.8% of total fatty acids). Brazilian raisin juice forms 72.6% of the weight of its fruits; the level of its titratable acidity is 0.9–1.0%, pH is 3.79–3.86, and it is characterized by a relatively high content of polyphenols.

With antioxidants, vitamins, minerals and fibre, consuming the raisin can be beneficial to the human body. In addition, phosphorus content is much higher than in other fruits.

Among other biologically active substances, *Physalis* contains withanolides, which are steroid lactones with a broad spectrum of medicinal properties. It is believed that these ingredients have liver-protective, anti-tumour, cytotoxic, anti-inflammatory, antibacterial, and hypotensive (blood pressure lowering) properties; they prevent brain stroke, are antifeedants and repellents, and also exhibit a calming effect [6,7].

*Physalis* is a thermophilic plant. It copes well with short-term dips in temperature, has relatively low susceptibility to diseases and pests, and has high productivity in terms of fruit number. Its shoots develop dichotomically, each fork having one flower and then fruit. From the literature it appears that one plant can produce up to 300 fruits [3]. With this productivity, fruit yield of 10–30 tonnes from 1 ha can be achieved. *Physalis* enters the period of flowering and fruiting relatively late. Fruiting occurs in the second half of summer, so that harvesting is in late August and September. In Polish climatic conditions thermophilic plants are grown from seedlings, often under cover (flat covers, plastic tunnels), with mulching of the soil. Of great importance for the profitability of production is the time of setting the plantation. On the basis of two years of observation, it can be concluded that this plant is slightly less sensitive to low temperatures under short-term frost than the tomato.

*Physalis* is sold fresh and dried, often under the name Inca berries or golden berries. The essence of the drying of food products is to be as quick as possible to remove moisture at a temperature that does not significantly change the characteristics of the product. Removal of water from agricultural raw materials is associated with a number of positive as well as negative consequences. The benefit of a dried product is its stability at room temperature, allowing for long-term storage and reduction of the weight and volume, which is relevant for transport and storage. Also, some technological properties, affecting further processing (e.g., milling of cereals), are improved. Adverse changes relate to the internal structure, which is decisive for the texture and firmness, sensory properties, hardness, taste, colour and smell, as well as the chemical composition affecting the contents of vitamins and nutrients.

The use of different drying techniques results in a variety of mechanisms for removing water and the conditions for running processes. This leads to obtaining products with extremely different characteristics [8,9,10]. The most commonly used drying technique is convective drying, consisting of exchange of moisture between the dried raw material and the hot air flowing through the drying chamber [11,12,13]. However, there is a widespread perception that it is a method that affects the dried material negatively, resulting in obtaining dried material with significantly reduced quality compared to products obtained by other drying techniques. The benefits are related to the low cost of manufacture of the product and a simple design and easy operation of the drying devices.

Assessment of the process of acquiring drying products should be carried out on the basis of four criteria: the speed of the flow, energy efficiency, the cost of the product and its quality [14]. The primary factor in the lowering of the quality of the product obtained in the form of dried material is the temperature at which the drying process is conducted. High process temperatures occur using a traditional convective method Modern drying techniques allow biological materials to be dried at lower temperatures and at the same time they allow the duration of the process to be shortened [15,16,17]. One technique for satisfying the above requirements is drying with microwave heating. Microwaves allow heat to be provided to the whole volume of the sample, which results in significant shortening of the duration of dehydration compared to conventional methods [18,19]. Additional use of lowered pressure in the drying chamber will reduce the process temperature, which significantly raises the product’s quality [20,21].

## 2. Results and Discussion

### 2.1. Effect of Processing on the Rheological Properties

**Figure 1:**
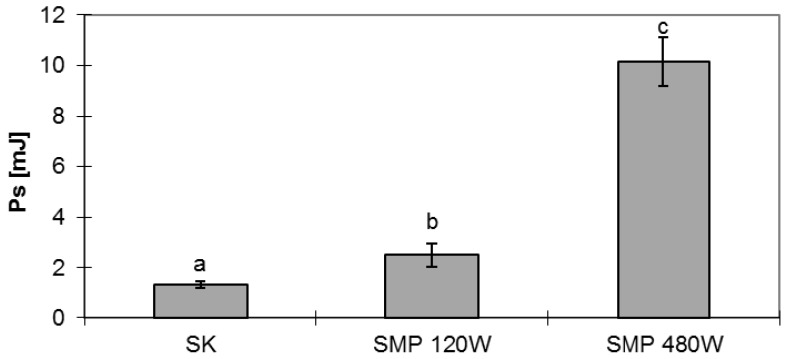
Values of compression work for dried *Physalis.* SK—convective method; SMP—microwave method under reduced pressure 120 W and 480 W; Mean values with different letters (a, b, c) within the same columns are statistically different (*p* = 0.05).

**Figure 2:**
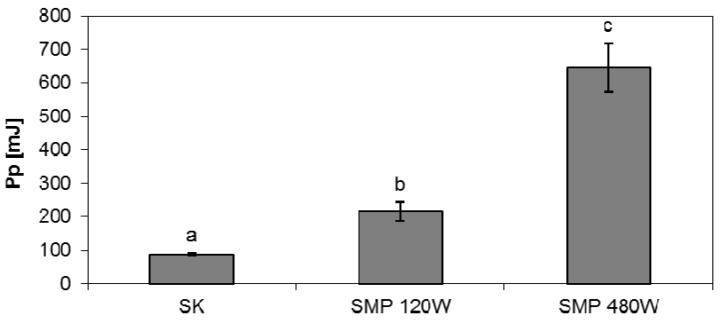
Values of work needed to cut the dried *Physalis.* SK—convective method; SMP—microwave method under reduced pressure 120 W and 480 W; Mean values with different letters (a, b, c) within the same columns are statistically different (*p* = 0.05).

**Figure 3:**
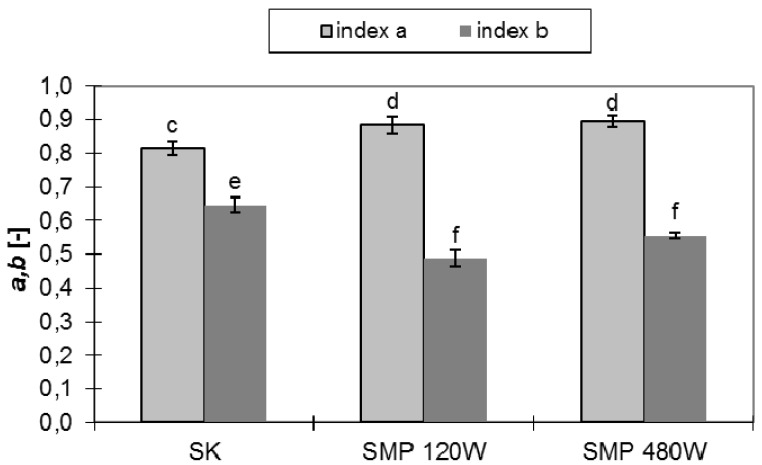
Values of a and b indexes for dried *Physalis.* SK—convective method; SMP—microwave method under reduced pressure 120 W and 480 W; Mean values with different letters (a, b, c) within the same columns are statistically different (*p* = 0.05).

Temperature of the microwave-dried material under reduced pressure during dewatering with a microwave power of 120 W did not exceed 70 °C, while at a microwave power of 480 W the material reached a temperature of 90 °C. Such temperatures are acceptable for microwave heating [22]. Work of compression is a physical property that informs us of the energy spent during deformation of the test material. This is a very important parameter, both from the point of view of further processing of the dried *Physalis*, as well as with regard to the sensory evaluation of the products intended for human consumption. The results of statistical analysis are presented in Figure 1, Figure 2 and Figure 3. Figure 1 shows the values of work done while compressing the *Physalis* being dried.

#### 2.1.1. Resistance to Compression

*Physalis* microwave dried under reduced pressure is characterized by higher resistance to compression than *Physalis* dried by the convection method. This is related to humidity of the dried material. Dehydration by the convection method lasts about five hours, and in spite of that the dried material had significantly higher humidity than the product subjected to microwave drying under reduced pressure. Moisture content is an essential factor determining the mechanical and rheological characteristics of the test materials [9]. A four-fold increase in power during microwave drying under reduced pressure also caused about 4-fold increase in the value of the work of compression. This may result from an increase in compactness of the dried material obtained during drying with higher power microwaves. The sensory evaluation of such materials as pumpkin and avocado indicates that droughts intended for consumption, which are characterized by higher compression resistance, have a higher sensory evaluation in terms of hardness, which means that a harder drought is more desirable [23,24].

#### 2.1.2. Cutting

Similar dependencies were obtained from the analysis of the values of cutting work (Figure 2). Dried materials obtained by the convective method require lower energy inputs to the process of cutting than those obtained by microwave heating at reduced pressure in the drying chamber. A microwave power surge causes a significant increase in the value of the work needed to cut dried material. This is related to changes in cellular structure caused by higher power microwaves, resulting in a smaller cell surface and thicker, more solid cell walls [22]. The loss of water in the drying material results in structural changes and hardening of the tested products. A similar phenomenon has been observed by other researchers [25,26].

#### 2.1.3. Elasticity

Flexibility is among the most important indicators to take into account in sensory evaluation of products intended for human consumption. Figure 3 shows the values of the indexes describing the process of stress relaxation for dried fruits. *Physalis* microwave-dried under reduced pressure is characterized by greater elasticity than the dried material obtained by the convective method, due to the significantly lower values of the (b) index. This means that the speed of stress decline in the relaxation test of material dried by microwave is lower. This also attests to less destructive changes to the internal structure of the materials being dehydrated by microwave heating. This is consistent with the opinions of other scholars claiming that convective drying is one of the techniques which most destructively affects the raw material. Some researchers suggest that plant tissue should be treated as a group of cells surrounded by a cell wall with visco-elastic properties [27]. The middle plate connecting neighbouring cells may be a type of “damper” that alleviates the effects of external influences. The state of conservation of the central plate, after the drying process, may determine the rheological properties of the drought.

### 2.2. Effect of Processing on the Chemical and Physical Properties

The assays of the content of polyphenols, antioxidant activity, activity of water, carotenoids, and colours were carried out for fresh *Physalis* treated as a control test, as well as for the three methods of drying. The results are summarized in Table 1, Table 2 and Table 3.

#### 2.2.1. Water Activity

Water activity is determined in order to specify the susceptibility of a foodstuff for the development of micro-organisms. Chemically pure water has an activity a_w_ = 1. Along with an increase in the concentration of dissolved compounds the activity of water falls. In the test-dried materials the activity was quite low, ranging from 0.232 to 0.524. It was lowest in material dried by microwave power of 480 W and the highest for conventional drying. With such low activity, none of the tested materials require any additional protection against the development of micro-organisms. The fresh fruit sample had water activity of 0.987, at which all micro-organisms have favourable conditions for development. Such high water activity (0.985–0.989) for fresh fruit of *Physalis* is confirmed by a study presented by Puente et al. [2]. Most micro-organisms develop water activity of above 0.95. The lowest water activity at which there is development of micro-organisms is 0.6. In the Vásquez-Parra et al. [28] study on drying *Physalis* under different conditions of water activity it was much higher, ranging from 0.58 to 0.89.

#### 2.2.2. Polyphenol Content

The fruits of *Physalis* are rich in polyphenols [2,4]. The content of total polyphenols significantly increased during drying in fresh fruit, being measured at 29.5 mg of gallic acid per 100 grams of dry mass (GA·100 g^−1^ DM). When dried conventionally it soared to 177.6 mg GA·100 g^−1^ DM, and the highest was recorded with drying by the microwave power of 480 W, reaching 436.3 mg GA·100 g^−1^ DM (Table 1). Evaporation of water during the drying process results in densification of the active ingredients, per unit of dry matter. During conventional drying, the high temperature and presence of oxygen cause partial oxidation of bioactive compounds. On the other hand, during microwave drying, the process is intensified and the drying time is reduced. This results in a reduction in the oxidation of polyphenols.

#### 2.2.3. Antioxidant Activity

When determining the antioxidant activity with the ferric-reducing antioxidant power (FRAP) method, there was an increase for each of the methods of drying and, similarly to the previous cases, the largest increase was for microwave drying at 480 W, and the smallest was by conventional drying. Similar results were obtained for determination of activity with the ABTS (2,2’-azino-bis(3-ethylbenzothiazoline-6-sulphonic acid) method. Polyphenols are known to have strong antioxidant activities [29] and there is a significant correlation between phenolic concentration and free radical scavenging activity [30]. Also, carotenoids have numerous bioactive properties—antioxidative, anti-inflammatory and anticancer [31].

#### 2.2.4. Carotenoid Content

There were five carotenoids identified in *Physalis*: all-*trans*-lutein, β-cryptoxanthin, α-carotene, all-*trans*-β-carotene and 15-*cis*-β-carotene (Table 2). The highest amount of α-carotene (190.15 mg·kg^−1^ DM) was observed at 480 W microwave drying. All-trans-lutein content in all four analysed variants was at the lowest level (6.61–13.87 mg·kg^−1^ DM) as compared to the other carotenoids. In fresh *Physalis* 15-*cis*-β-carotene was not detected. Total carotenoid content was detected at the lowest level in fresh *Physalis* (168.25 mg·kg^−1^ DM) and in fruits after conventional drying (169.85 mg·kg^−1^ DM). There were no statistical differences between these two values. Similar relations were obtained by Nawirska et al. [32] for drying of pumpkin with different methods.

#### 2.2.5. Colour Assessment

The dry materials obtained were characterized by bright colour, which was brightest for microwave drying at 480 W. From the data presented by Puente et al. [2] it appears that the brightness of fresh *Physalis* was much lighter (lightness (L*) = 70.31–71.37) than that used for drying in the present studies (L* = 43.28). However, in research presented by Vásquez-Parra et al. [28] the brightness of fresh *Physalis* was greater (L* = 38.61), and in the course of drying the fruit brightness became greater, which is in accord with the research presented in this article. Conventional drying resulted in a slight dimming of tested fruit.

Conventional drying caused significant browning of the *Physalis* tested, while microwave drying at 480 W resulted in a more yellow colour (Table 3). In the research by Vásquez-Parra et al. [28] the parameter b* (yellow/blue opponent colors) for fresh *Physalis* was 37.37, while after drying it was in the range of 26.74–39.86. In our studies, this parameter for microwave drying at 480 W was significantly higher (52.82).

High levels of phenolic compounds were reported for the cape gooseberry fruit, and enzymatic browning (i.e., the action of polyphenol oxidase (PPO) over phenolic compounds in presence of oxygen) could also explain the browning of fruits after drying.

## 3. Materials and Methods

### 3.1. Materials

In the experiment conducted in 2015 the effect of drying methods on quality of *Physalis* fruit was evaluated. *Physalis peruviana* was grown in the Experimental Station of The Wrocław University of Environmental and Life Sciences. Eight-week-old transplants produced in the greenhouse were planted on plots fertilized with nitrogen (150 kg N·ha^−1^) in the second decade of May and fruits were harvested at the end of September. Fruits were harvested in technological maturity. The dry mass of fresh fruit was 20.9%.

### 3.2. Drying Methods

*Physalis* fruit was dried with two methods: convective (SK) and by microwave under reduced pressure (SMP). Preliminary studies on dehydrating *Physalis* showed that the fruit is hard to dehydrate, which is due to the thick and relatively hard skin which effectively limits the penetration of heat into the interior of the material. High temperature and fast flow of the drying agent during convective drying can bring the interior to the boiling point, but the skin is a barrier limiting evaporation. Inside there is high pressure causing a kind of explosion of the fruits. This is not a negative phenomenon due to the limited use of such dried material. The solution to the problem is the use of microwave heating in order to facilitate the evaporation of moisture from the inside of the fruit, after executing a series of punctures in the peel using a penetrator [33].

From the fruit delivered to the laboratory, the smallest pieces, which were immature or mechanically damaged, were removed. The rest of the fruits were subjected to homogeneous pricking on the entire surface with a penetrator 1 mm in diameter. Each fruit was punctured 10 times. The prepared fruits were dried convectively, using a prototype convection chimney dryer installation (Twoja energia, Warsaw, Poland) in a single layer at the air temperature of 70 °C and the air flow velocity of 1.5 m·s^−1^. Many researchers have confirmed that the drying agent temperature of 70 °C is the maximum for biological materials, while the air flow rate of 1.5 m·s^−1^ reduces the drying time [34]. Microwave drying under reduced pressure was performed under the following conditions: microwave power of 120 and 480 W, amplitude control of magnetrons, and pressure in the drying chamber of 4–10 kPa. The power range of the microwave was determined based on literature analysis and prior author research. The power value of 120 W is the lowest that can be obtained by the magnetrons used in the installation. Use of power above 480 W often leads to a partially burnt drought. The vacuum range of 4–10 kPa allowed for drought, with a minimum puffing effect. The SM 200 dryer from Plazmatronika, Wrocław, Poland [19,20] was used for microwave drying under reduced pressure. The raw material was dried to the equilibrium humidity. Three technological repetitions for each variant of the drying were made.

### 3.3. Mechanical Strength and Rheological Studies

Mechanical strength and rheological studies were executed on an Instron 5566 machine equipped with a strain gauge head of 0.5 class and maximum load of 100 N. Compression tests were performed on single samples, which were deformed by 20% of the initial height. The pressing slab moved at 1.8 mm·min^−1^ [32]. Measurements were made in 10 repetitions, from which the work of compression was calculated (Ps). Cutting tests were made for individual fruits. Use was made of a specialized knife made by the Instron, blade, with opening angles of 60°. Knife penetration speed was 10 mm·min^−1^, allowing a complete severing of the sample. Measurements were made in 10 repetitions. The initial stress for recording the relaxation process was defined at a level corresponding to the stress in the material deformed by 20% of its initial height. The first phase of the process was conducted with the compression plate moving at 10 mm·min^−1^. The measurements were done in 5 repetitions. The method, used in the study, of comparing the relaxation curves for different materials allows us to unambiguously assess how the technological treatment affects the elasticity of the derived product [20]. Index (a) specifies the level to which the stress falls during the process of stress relaxation. It assumes values in the range from 0 to 1. For a = 0 stress relaxation does not occur, which means that we are dealing with a perfectly flexible body. Index (b) informs us about the speed of stress decay during the test. The closer to zero the (b) value, the more elastic is the body.

### 3.4. Water Activity

Chemical characterization of the dried materials included determination of water activity in them. This study was carried out on LabMaster apparatus for testing water activity made by Novasina of ± 0.003 precision at 25 ± 1.5 °C.

### 3.5. Carotenoid Content by Ultra Performance Liquid Chromatography-Photodiode Detector-Mass Spectrometry (UPLC−PDA−MS) Analysis

For the extraction and determination of carotenoids, a protocol described earlier was followed [35]. The powder samples of fruits (0.25 g) containing 10% MgCO_3_ were continuously shaken with 5 mL of hexane:acetone:methanol (2:1:1, *v*/*v*/*v*) containing 1% of Butylhydroxytoluene (BHT), at 500 rpm (DOS-10L Digital Orbital Shaker, Elmi Ltd., Riga, Latvia) for 30 min in the dark. After the first extraction, the samples were centrifuged at 19,000× *g* for 10 min at 4 °C, and the supernatant was recovered. The samples were re-extracted and centrifuged in the same conditions. Supernatants were combined and evaporated to dryness. The pellet was re-extracted using 2 mL of 100% methanol, filtered through a hydrophilic polytetrafluoroethylene (PTFE) 0.20-µm membrane (Millex Samplicity Filter, Merck, Darmstadt, Germany) and used for analysis.

Compounds were separated with an ACQUITY UPLC BEH RP C18 column (1.7 µm, 2.1 mm × 100 mm, Waters Corp, Milford, MA, USA.) at 32 °C. The elution solvents were ACN:MeOH (7:3, *v*/*v*) (A) and 0.1% formic acid (B). Samples (10 µL) were eluted according to the linear gradient described previously [35]. Weak and strong needle solvents were ACN–MeOH (7:3, *v*/*v*) and 2-propanol, respectively.

Identification of carotenoids was carried out on the basis of fragmentation patterns and on the basis of photodiode array (PDA) profiles. Where available, compounds were compared with authentic standards (their fragmentation pathways, retention times and PDA profiles). The runs were monitored at 450 and 427 nm. The PDA spectra were measured over the wavelength range of 200–800 nm in steps of 2 nm. Calibration curves were made from all-*trans*-β-carotene and all-*trans*-lutein. All incubations were done in triplicate. The results were expressed as milligrams per kilogram of dry matter (DM).

### 3.6. Analysis of Polyphenols and Determination of Antioxidant Activity

Samples for the analysis of polyphenols and antioxidant activity (ABTS, FRAP) were prepared as follows. Approximately about 0.5 g of each cape gooseberry fruit was weighed into a test tube for antioxidant property analysis. A total of 15 mL of 80% aqueous ethanol was added, and the suspension was stirred. The sample was sonicated for 20 min, and left at 4 °C for 24 h. (ultrasonic device, Sonic 6D, Warsaw, Poland). After this time the extract was centrifuged at 12,500 rpm for 6 min, and the supernatants were recovered.

Total polyphenols were determined by the Folin–Ciocalteu [36] and described previously [37].

*ABTS^+^ radical scavenging spectrophotometric assay.* The ABTS^+^ radical scavenging activity of the sample was measured according to the method developed by Re et al. [38] and described previously [37].

*Ferric-reducing antioxidant power (FRAP) assay.* The reducing potential of the sample was determined using the FRAP assay, proposed by Benzie and Strain [39] and described previously [37].

### 3.7. Physalis Colour Assessment

The colour of dried materials was determined using the CIELab system. The colour of the gooseberry fruits was measured with ColorQuest XE (Hunter Lab). Milled fruits were placed in a glass cuvette, and the colour was recorded using CIE L*a*b* 10°/D65 colour spaces, where L* indicates lightness, a* is the +a redness, and b* is the +b yellowness.

### 3.8. Statistical Analysis

The data obtained were subjected to statistical analysis performed with Statistica 9.0 (StatSoft, Krakow, Poland). They were recorded as means ± standard deviation (SD), and analysed by Excel 2007. Analysis of variance was performed by analysis of variance (ANOVA) procedures. Significant differences (*p* ≤ 0.05) between the mean values were determined by Duncan’s multiple range test.

## 4. Conclusions

Microwave drying was definitely better with respect to the analysed qualitative effects on the *Phisalis peruviana*. The best results were obtained for drying using microwaves of 480 W. Dried materials obtained in this way were characterized by higher contents of bioactive compounds of the best antioxidant properties, and at the same time the smallest water activity. They were also the brightest and had a smooth yellow colour, attractive for the consumer.

## Figures and Tables

**Table 1:** Content of water activity, total polyphenols and antioxidant activity in the fruit of *Physalis peruviana* with different methods of drying.

Treatments-Drying Methods	Water Activity-	Polyphenols mgGA·100 g^−1^	FRAP mmol Trolox·100 g^−1^	ABTS mmol Trolox·100 g^−1^
Fresh berry	0.987 ± 0.09 ^a^	29.5 ± 0.9 ^d^	0.19 ± 0.02 ^d^	0.72 ± 0.24 ^d^
Conventional drying	0.524 ± 0.03 ^b^	177.6 ± 0.7 ^b,c^	0.86 ± 0.25 ^c^	5.30 ± 0.52 ^c^
Microwave at 120 W	0.304 ± 0.04 ^c^	228.7 ± 3.3 ^b^	1.61 ± 0.19 ^b^	9.71 ± 1.03 ^b^
Microwave at 480 W	0.232 ± 0.02 ^c,d^	436.3 ± 2.4 ^a^	1.91 ± 0.18 ^a^	19.06 ± 1.12 ^a^

Mean values with different letters (a–d) within the same column are statistically different (*p* = 0.05) Values are expressed as the mean ± standard deviation. FRAP: ferric-reducing antioxidant power. mgGA: miligrams of gallic acid. ABTS: 2,2’-azino-bis(3-ethylbenzothiazoline-6-sulphonic acid).

**Table 2:** Content of carotenoids in fruit of *Physalis peruviana* with different methods of drying.

Treatments-Drying Methods	All-*trans*-Lutein	β-Kryptoxantin	α-Carotene	All-*trans*-β-Carotene	15-*cis*-β-Carotene	Total Carotenoids
	mg·kg^−1^ DM					
Fresh berry	6.61 ± 0.08 ^d^	23.22 ± 0.15 ^b^	111.79 ± 11.02 ^c^	26.62 ± 1.98 ^c^	ND	168.25 ± 11.21 ^c^
Conventional drying	7.04 ± 0.01 ^c^	14.36 ± 0.16 ^c^	114.17 ± 11.56 ^c^	27.58 ± 2.11 ^c^	6.70 ± 0.06 ^c^	169.85 ± 12.54 ^c^
Microwave at 120 W	7.85 ± 0.02 ^b^	12.29 ± 0.12 ^d^	176.25 ± 12.54 ^b^	47.74 ± 3.78 ^a^	11.86 ± 0.37 ^b^	255.98 ± 15.28 ^b^
Microwave at 480 W	13.87 ± 0.15 ^a^	29.23 ± 0.18 ^a^	190.15 ± 21.89 ^a^	34.61 ± 2.84 ^b^	29.08 ± 1.05 ^a^	296.94 ± 22.37 ^a^

ND: not detected; DM, dry mass. Mean values with different letters (a–d) within the same column are statistically different (*p* = 0.05) Values are expressed as the mean ± standard deviation.

**Table 3:** Colour of dry *Physalis peruviana.*

Treatments-Drying Methods	L*	a*	b*
Fresh berry	43.28 ± 1.21 ^c^	6.98 ± 0.09 ^c^	29.77 ± 0.94 ^c^
Conventional drying	40.66 ± 0.54 ^d^	6.15 ± 0.7 ^c^	19.41 ± 0.71 ^d^
Microwave at 120 W	59.36 ± 8.94 ^b^	8.33 ± 0.19 ^b^	32.03 ± 0.85 ^b^
Microwave at 480 W	62.89 ± 7.89 ^a^	15.47 ± 0.51 ^a^	52.82 ± 0.62 ^a^

Mean values with different letters (a–d) within the same column are statistically different (*p* = 0.05) Values are expressed as the mean ± standard deviation. L*: lightness. a*: The red/green opponent colors, with green at negative a* values and red at positive a* values. b*: The yellow/blue opponent colors, with blue at negative b* values and yellow at positive b* values.
